# Association between Regular Electronic Nicotine Product Use and Self-Reported Periodontal Disease Status: Population Assessment of Tobacco and Health Survey

**DOI:** 10.3390/ijerph16071263

**Published:** 2019-04-09

**Authors:** Nkiruka C. Atuegwu, Mario F. Perez, Cheryl Oncken, Sejal Thacker, Erin L. Mead, Eric M. Mortensen

**Affiliations:** 1 Department of Medicine, UConn Health, University of Connecticut School of Medicine, Farmington, CT 06030, USA; atuegwu@uchc.edu (N.C.A.); maperez@uchc.edu (M.F.P.); oncken@uchc.edu (C.O.); mead@uchc.edu (E.L.M.); 2 Division of Periodontology, UConn Health, University of Connecticut School of Dental Medicine, Farmington, CT 06030, USA; sthacker@uchc.edu

**Keywords:** electronic nicotine product, e-cigarette, periodontal disease, oral disease, oral health

## Abstract

Electronic nicotine product use is increasing in the U.S., but few studies have addressed its effects on oral health. The goal of this work was to determine the association between electronic nicotine product use and periodontal disease. Population Assessment of Tobacco and Health adult survey data from 2013–2016 (waves 1, 2 and 3) was used for the analysis. Longitudinal electronic nicotine product users used electronic nicotine products regularly every day or somedays in all three waves. Participants with new cases of gum disease reported no history of gum disease in wave 1 but reported being diagnosed with gum disease in waves 2 or 3. Odds ratios (OR) were calculated to determine the association between electronic nicotine product use and new cases of gum disease after controlling for potential confounders. Compared to never users, longitudinal electronic nicotine product users had increased odds of being diagnosed with gum disease (OR 1.76, 95% Confidence Interval (CI) 1.12–2.76) and bone loss around teeth (OR 1.67, 95% CI 1.06–2.63). These odds were higher for participants with a history of marijuana and a history of illicit or non-prescribed drug use. Our findings show that e-cigarettes may be harmful to oral health.

## 1. Introduction

Electronic nicotine product use is increasing in the United States [1,2] but few studies have addressed their toxicological effects on oral health [3]. Electronic nicotine products, commonly known as e-cigarettes, include a diverse group of battery-powered devices that allow users to vaporize and inhale an aerosol, which usually contains nicotine, flavorings, and other additives. These products vary in design and appearance, can sometimes be called other names such as e-cigars, e-pipes, e-hookahs, personal vaporizers, vape pens and hookah pens, but they generally contain similar components and operate in a similar manner [2].

Periodontal (or gum) disease is an infectious disease resulting in inflammation within the supporting tissues of the teeth, and progressive diminished tooth attachment and bone loss. It is characterized by pocket formation and/or gingival recession. Periodontal disease is a significant cause of tooth loss in the United States [4]. It also ranks among the top 100 causes of disability-adjusted life years globally [5]. Smoking is a major risk factor for periodontal disease [6] and may be responsible for more than half of periodontal disease cases among adults in the United States [7]. In vitro studies have shown that e-cigarette vapor can lead to inflammation of gingival epithelial cells similar to that observed in cells exposed to conventional cigarette smoke [8].

There have been numerous studies showing the deleterious effects of periodontal disease on overall health. Periodontal disease is associated with an increase in cardiac disease among individuals >65 years of age [9], exacerbation of insulin resistance [10,11] and increase in the risk of cardiorenal mortality for those with type 2 diabetes [12]. Adults with periodontal disease were more likely to have chronic kidney disease [13] and also a greater risk of mortality [14,15]. Periodontal disease has also been shown to lead to a significant increase in the risk of oral cancer [16], a decrease in the cognitive function for the elderly [17,18] and an accumulation of amyloid β plaques which is a central feature of Alzheimer’s disease [19].

Very few studies have investigated the effects of e-cigarette use on oral health, and most of these studies have been limited by few participants. One recent study suggested that e-cigarette use may not be as hazardous to periodontal health as cigarette smoking, and e-cigarette users may have periodontal status comparable to non-smokers [20]. Another study showed a progressive improvement in the periodontal indexes for cigarette smokers who switched to e-cigarettes for approximately 4 months before the study [21]. However, another study showed a statistically significant increase in gingival inflammation in tobacco smokers who switched from smoking to vaping for two weeks [22]. A larger cross-sectional analysis using the 2016 Behavioral Risk Factor Surveillance System survey data showed an increased odds of teeth loss due to decay or gum disease with the use of e-cigarettes [23].

The aim of this study was to examine the association between electronic nicotine product use and new cases of gum disease in a large, nationally representative sample, using data from the Population Assessment of Tobacco and Health (PATH) study. The hypothesis was that the use of electronic nicotine products would be associated with increased odds of gum disease and bone loss around teeth, even after controlling for use of conventional cigarettes and other known risk factors. Sub-group analysis was also performed to examine this association in participants who had a history of marijuana use and a history of illicit or non-prescribed drug use.

## 2. Material and Methods

Public use PATH survey data from 12 September 2013 to 14 December 2014 (wave 1), 23 October 2014 to 30 October 2015 (wave 2), and 19 October 2015 to 23 October 2016 (wave 3) was used for the analysis [24]. The PATH survey is a nationally representative, longitudinal cohort study of tobacco-use patterns and the health of non-institutionalized adults (≥18 years) and youths (12–17 years) in the United States. The PATH survey collects interview data through in-person computer-assisted personal interviewing and audio computer-assisted self-interviewing methods. All wave 1 respondents were eligible for the wave 2 and 3 interview as long as they continued to live in the United States and were not incarcerated. There were 32,320 adults in wave 1, 28,362 adults in wave 2, and 28,148 adults in wave 3. The 23,670 adults that completed waves 1, 2 and 3 interviews were used for the study. Weights were used to compensate for differential nonresponse rates, variable probabilities of selection and possible deficiencies in sampling. More details about the PATH study design and methods are published elsewhere [25].

### 2.1. Inclusion Criteria

We included the following participants in our analysis:

(1) Adults who completed Waves 1, 2 and 3 of the PATH survey.
(2) Participants who reported ever having had their teeth cleaned by a dentist, hygienist, or other health professionals by wave 3.
(3) Participants who reported no history of “gum disease” at baseline (wave 1).

### 2.2. Data and Definitions

Data was collected on the age, sex, race, highest grade completed, income level, current tobacco use, current second-hand exposure to tobacco smoke (cigarettes, cigars, filtered cigars or cigarillos), use of nicotine replacement therapy products, marijuana use, alcohol use, illicit and non-prescribed drug use, visits to the dentist and medical history as of wave 1.

Participants with a history of marijuana use in wave 1 reported “ever smoking part or all of a cigar, cigarillo or filtered cigar with marijuana in it” or “having ever used marijuana, hash, THC or grass”. Participants with a history of illicit drug use in wave 1 reported ever having used any of the following substances: cocaine or crack, stimulants like methamphetamine or speed or drugs like heroin, inhalants, solvents, or hallucinogens. Participants with a history of non-prescribed drug use in wave 1 reported ever having used painkillers, sedatives, tranquilizers, Ritalin or Adderall not prescribed to them or using the drugs “for the experience or feeling they caused”.

### 2.3. Longitudinal Product Use

Regular electronic nicotine product users were participants who said yes to using “electronic nicotine products (such as e-cigarettes, vape pens, personal vaporizers and mods, e-cigars, e-pipes, e-hookahs and hookah pens) fairly regularly every day or some days. Longitudinal electronic nicotine product users were participants who were regular electronic nicotine product users in all the three waves of the PATH survey. Longitudinal conventional cigarette users were participants who have smoked more than 100 cigarettes in their lifetime and smoked every day or someday in all the three waves of the PATH survey.

### 2.4. Outcomes

Participants with new cases of gum disease reported having been told by a dentist, hygienist, or other health professionals in the past 12 months that they had “gum disease” in wave 2 or 3 while also reporting no history of “gum disease” in wave 1. Participants with bone loss around teeth reported having ever been told by a dentist, hygienist, or other health professionals that they lost bone around their teeth in wave 3 (this question was first added to the survey in wave 3). Participants with any periodontal disease reported bone loss around teeth or new cases of gum disease in wave 2 or 3.

### 2.5. Statistical Analyses

Descriptive statistics of never electronic nicotine product, longitudinal electronic nicotine product and non-longitudinal electronic nicotine product users’ demographics, tobacco use and medical history were calculated. Non-longitudinal electronic nicotine product users comprise of ever electronic nicotine product users that did not use electronic nicotine product regularly every day or somedays in waves 1, 2 and 3. Continuous variables were analyzed using Student’s t-test and categorical variables were analyzed using chi-square test (Table 1).

Multivariable logistic regression was used to examine the association between longitudinal electronic nicotine product use and new cases of gum disease, bone loss around teeth and any periodontal disease after controlling for the confounding factors listed in Table 1. All the covariates in Table 1 were included in the multivariable regression models.

For the secondary analysis, multivariable logistic regression was also used to examine the association between longitudinal electronic nicotine product use and new cases of gum disease, bone loss around teeth and any periodontal disease in two subgroups. The subgroups were participants that had a history of marijuana use and a history of illicit or non-prescribed drug use. Confounding factors listed in Table 1 were also controlled for in the analysis.

Replicate weights and balanced repeated replication methods were used for the analysis in order to account for the PATH study design. Participants with missing values were removed from the analysis. All analyses were conducted using R version 3.4.2 (R Core Team, Vienna, Austria). Statistical significance was defined as a two-tailed *p*-value of < 0.05.

## 3. Results

**Figure 1:**
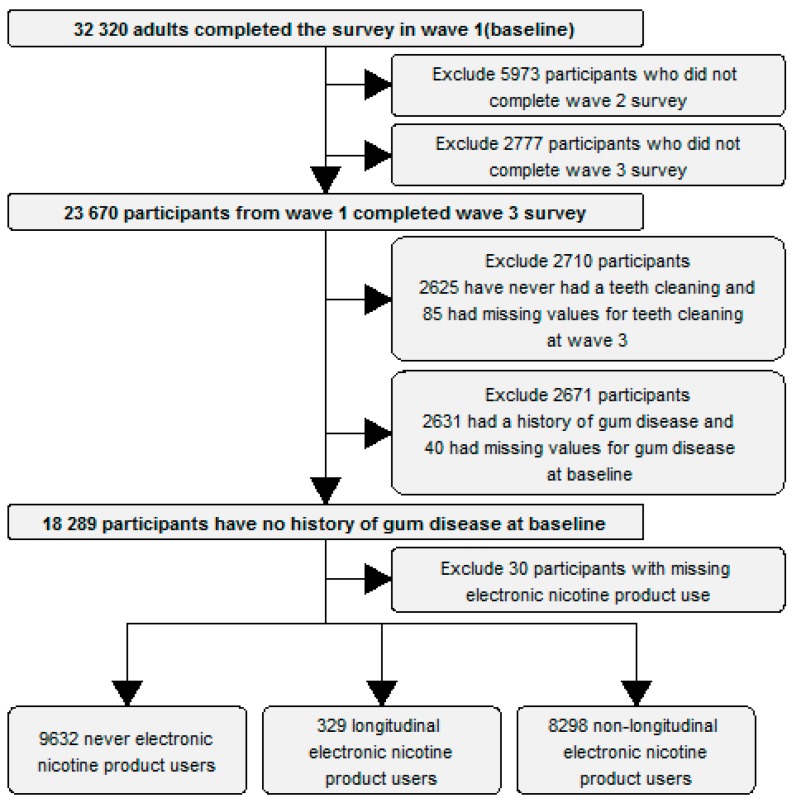
Flow chart showing selection criteria.

A total of 18,289 participants who met the inclusion criteria were included in the analysis. Figure 1 shows the selection process for the participants. There were 329 participants who reported longitudinal electronic nicotine product use, 8298 participants who reported non-longitudinal electronic nicotine product use and 9632 participants who reported never electronic nicotine product use. Table 1 shows the descriptive statistics for the baseline characteristics and outcomes for participants used in the analysis.

Among the participants with no gum disease at baseline, 46.3% (95% CI 45.8 – 46.9) were men, 0.9% (95% CI 0.8–1.0) were longitudinal electronic nicotine product users, 23.6% (95% CI 22.9–24.4) were non-longitudinal electronic nicotine product users, 75.5% (95% CI 74.7–76.2) were never electronic nicotine product user and 13.0% (95% CI 12.5–13.5) were longitudinal conventional cigarette users. Among participants with no gum disease at baseline, 5.4% (95% CI 5.0–5.8) reported new cases of gum disease at waves 2 and 3, 8.2% (95% CI 7.6–8.9) reported ever having bone loss around teeth at wave 3 and 11.3% (95% CI 10.9–12.4) reported any periodontal disease at waves 2 or 3.

Longitudinal electronic nicotine product users had increased odds of being diagnosed with gum disease (OR 1.76, 95% CI 1.12–2.76), bone loss around the teeth (OR 1.67, 95% CI 1.06–2.63) and any periodontal disease (OR 1.58, 95% CI 1.06–2.34) compared to never-electronic nicotine product users after adjusting for longitudinal cigarette use and other confounding factors. The odds of being diagnosed with gum disease and bone loss were slightly higher for participants that had a history of marijuana use and a history of illicit or non-prescribed drug use (Table 2).

## 4. Discussion

We showed that participants with no history of gum disease who used electronic nicotine products regularly every day or somedays for a year or more had increased odds of being diagnosed with gum disease, even after controlling for conventional cigarette smoking and other known risk factors. The participants who used electronic nicotine products also had an increased odds of reporting bone loss around teeth which is indicative of advanced periodontal disease. The odds were higher for participants who had a history of marijuana use or any illicit and non-prescribed drug use. This study helps to fill a gap identified in the literature about the effect of electronic nicotine products use on oral health [26].

Marijuana use [27] and illicit drug use [28] have been implicated as risk factors for periodontal disease. Therefore, it is reasonable for our findings to show that these groups have higher odds of having periodontal disease with the use of electronic nicotine products. Our study shows that the co-use of electronic nicotine products with marijuana or illicit or non-prescribed drugs may amplify the risk of periodontal disease.

In vitro studies support the association that we found between electronic nicotine product use and periodontal disease. E-cigarette vapor has been shown to promote cell apoptosis, necrosis [8] and persistent DNA damage in gingival epithelium [29]. Menthol flavoring in e-cigarette liquid has also been shown to reduce the proliferation rate of human periodontal ligament fibroblasts [30]. Nicotine found in many e-cigarette aerosols [31] has been shown to inhibit the growth of gingival fibroblasts [32], human periodontal ligament cells [33] and alter the function of oral or peripheral neutrophils [34].

E-cigarettes have been perceived as less harmful than conventional cigarette smoking [35], and reported by some as a safer alternative to cigarette smoking [36] but e-cigarettes and other electronic nicotine products may still be deleterious to human health. Harmful or potentially harmful compounds such tobacco-specific nitrosamines, polycyclic aromatic hydrocarbons, metals and volatile organic compounds have been documented in some electronic nicotine product aerosols and the urine of e-cigarette users [1,37].

One strength of the study was the large number of participants available for the analysis, who were nationally representative of the United States, non-institutionalized adult population. Also the data was longitudinal, which allowed us to investigate the association between the use of electronic nicotine products and a diagnosis of gum disease and bone loss around the teeth for participants who had no reported history of gum disease. Some of the limitations of our study were the fact that the survey results could be affected by recall bias by the participants. There could also be diagnosis misclassification bias by the participants. “Regular use of electronic nicotine product” may be interpreted different by the participants. Ideally, quantifiable numbers such as electronic nicotine product use per week would have been interpreted better by the participants. Additionally, some of the participants may have had gum disease in wave 1 but were not aware of it. Also some participants with advanced periodontal disease such as bone loss around the teeth may have reported no gum disease in waves 2 or 3.

## 5. Conclusions

Our study with a nationally representative population indicates that electronic nicotine product use is associated with increased risk of developing periodontal disease even after accounting for the use of other tobacco products and other confounders. More research is necessary to assess the long-term effects and the safety of electronic nicotine products on oral health. Medical professionals should consider impact on oral health when educating and advising their patients about electronic nicotine product use.

## Figures and Tables

**Table 1:** Demographic characteristics and health history of adult electronic nicotine product users and controls at baseline (wave 1).

	Never Electronic Nicotine Product User N = 9632 % (95% CI)	Longitudinal Electronic Nicotine Product User N = 329 % (95% CI)	Non Longitudinal Electronic Product User N = 8298 % (95% CI)
**Age group**			
18 to 24 years old	9.6 (9.2–10)	23.8 (19.5–28.2)	30.8 (29.8–31.8)
25 to 34 years old	15.7 (14.8–16.6)	30.8 (24.4–37.1)	29 (27.8–30.3)
35 to 44 years old	17.4 (16.5–18.3)	15.9 (10.5–21.3)	16.6 (15.6–17.6)
45 to 54 years old	19.3 (18.5–20.1)	14.4 (9.5–19.3)	12.4 (11.6–13.3)
55 years old or older	38 (37–39)	15.1 (11.6–18.5)	11.1 (10.2–12)
**Gender**			
Female	55.6 (54.9–56.3)	46.8 (40.3–53.3)	47.7 (46.6–48.8)
Male	44.4 (43.7–45.1)	53.2 (46.7–59.7)	52.3 (51.2–53.4)
**Race**			
White	79.9 (79.1–80.7)	86 (82–90)	76.7 (75.3–78.1)
Black	11 (10.5–11.5)	5.3 (3–7.5)	13.3 (12.3–14.3)
Other	9.1 (8.5–9.7)	8.8 (5.4–12.1)	10 (9.1–10.9)
Hispanic	13.8 (13.2–14.3)	8.6 (5.6–11.6)	15.7 (14.7–16.7)
**Highest Grade of Education**			
Less than High School	9 (8.5–9.6)	5.4 (3.1–7.6)	9.9 (9.3–10.5)
General Education Diploma (GED)	3.6 (3.1–4)	5.9 (3.2–8.6)	7.6 (6.8–8.3)
High school graduate	23.1 (22.4–23.9)	20.3 (15.7–24.9)	25 (23.8–26.3)
Some college or associates degree	29.5 (28.8–30.1)	47.3 (40.8–53.8)	40.6 (39.3–41.9)
Bachelor’s degree or higher	34.8 (34.2–35.4)	21.1 (15.9–26.4)	16.9 (15.7–18)
Body Mass Index (mean)	28.0 (27.8–28.2)	27.8 (27.0–28.5)	27.5 (27.3–27.7)
**Income**			
Less than $10,000	9.8 (9–10.6)	13.5 (9.2–17.7)	18.8 (17.8–19.8)
$10,000 to $24,999	16.9 (15.8–18)	23 (18.1–28)	23.5 (22.2–24.7)
$25,000 to $49,999	22.2 (20.9–23.5)	26.9 (20.7–33.1)	24.1 (23–25.2)
$50,000 to $99,999	29.2 (27.7–30.7)	25.6 (20.1–31.1)	21.6 (20.4–22.9)
$100,000 or more	21.9 (20.5–23.3)	11 (7.2–14.9)	12 (10.9–13.1)
Baseline visit to the dentist ^1^	69.4 (68.2–70.6)	56.9 (51.3–62.4)	54.3 (52.8–55.8)
Longitudinal visit to dentist ^2^	76.4 (75.2–77.6)	67.3 (61–73.6)	63.5 (62.1–64.8)
History of prescription drug abuse	11.5 (10.7–12.3)	34.6 (28.4–40.7)	28.5 (27–29.9)
History of stomach, duodenal or peptic ulcer	7 (6.2–7.9)	9.4 (5.8–13)	6.7 (6.1–7.4)
History of respiratory disease ^3^	13.6 (12.7–14.5)	20.1 (16–24.2)	16.9 (16.1–17.7)
History of diabetes	13.7 (12.6–14.7)	6.7 (3.9–9.4)	8.4 (7.6–9.2)
History of high blood pressure	28.1 (27–29.2)	21.8 (17.2–26.4)	18.2 (17.1–19.3)
History of high cholesterol	24.4 (23.3–25.5)	13.8 (10.1–17.5)	12.8 (11.8–13.9)
Longitudinal conventional cigarette user	4.3 (4.0–4.6)	38.6 (32.1–45.1)	40.1 (38.7–41.4)
Former conventional cigarette user ^4^	20.5 (19.2–21.9)	38.6 (32.5–44.7)	14.5 (13.5–15.5)
History of marijuana use	28.5 (27–29.9)	70.2 (64.3–76.1)	66.3 (64.8–67.8)
History of alcohol use	80 (77.9–82.1)	91.4 (87.8–95)	90.6 (89.9–91.4)
History of other tobacco or tobacco product replacement therapy use ^5^	42.3 (40.7–43.8)	89.3 (85.3–93.3)	85.5 (84.3–86.7)
History of illicit drug use	9.2 (8.3–10.1)	29.8 (24.3–35.2)	25.7 (24.4–27)
History of current second hand exposure to tobacco smoke ^6^	14.8 (13.8–15.8)	39.3 (33–45.7)	41.4 (40–42.8)
**Outcomes**			
New cases of gum disease ^7^	5.1 (4.5–5.6)	9.8 (6.4–13.3)	6.2 (5.6–6.7)
Bone loss around teeth ^8^	8.4 (7.6–9.2)	11.2 (7.6–14.8)	7.3 (6.6–8.1)
Any periodontal disease ^9^	11.7 (10.8–12.6)	16.7 (12.2–21.2)	11.4 (10.6–12.2)

All the covariates in Table 1 were included in the multivariable regression models. ^1^ Saw a dentist in the past 12 months in wave 1. ^2^ Saw a dentist in the past 12 months in either wave 2 or wave 3. ^3^ Reported being told by a doctor or health professional that they had any of the following: COPD, chronic bronchitis, emphysema or asthma in wave 1. ^4^ Smoked more than 100 cigarettes in lifetime, and now does not smoke at all in wave 1. ^5^ Reported ever using any of the following products: Traditional or filtered cigars, cigarillos, pipe, hookah, smokeless tobacco, snus, dissolvable tobacco and nicotine patch, gum, inhaler, nasal spray, lozenge or pill in wave 1. ^6^ Reported living with someone that smokes cigarettes, cigars, cigarillos or filtered cigars in wave 1. ^7^ Reported new cases gum disease in wave 2 or 3. ^8^ Reported bone loss in wave 3. ^9^ Reported bone loss around teeth in wave 3 or new cases of gum disease in wave 2 or 3.

**Table 2:** Results of the Multivariable Logistic Regression Models.

	Odds Ratio (95% Confidence Interval)		
	New Cases of Gum Disease	Bone Loss around Teeth	Any Periodontal Disease ^1^
**Entire cohort (N = 18,259)**			
Never electronic nicotine product user	Reference	Reference	Reference
Longitudinal electronic nicotine product user	1.76 (1.12–2.76) ^2^	1.67 (1.06–2.63) ^2^	1.58 (1.06–2.34) ^2^
Non longitudinal electronic product user	1.09 (0.87–1.35)	1.10 (0.91–1.33)	1.09 (0.93–1.29)
**History of marijuana use (N = 9325)**			
Never electronic nicotine product user	Reference	Reference	Reference
Longitudinal electronic nicotine product user	1.95 (1.14–3.34) ^2^	1.91 (1.15–3.19) ^2^	1.91 (1.22–2.99) ^2^
Non longitudinal electronic product user	0.91 (0.69–1.21)	1.16 (0.90–1.50)	1.06 (0.86–1.30)
**History of illicit or non-prescribed drug use (N = 5410)**			
Never electronic nicotine product user	Reference	Reference	Reference
Longitudinal electronic nicotine product user	2.38 (1.33–4.26) ^2^	1.88 (1.01–3.48) ^2^	2.24 (1.40–3.60) ^2^
Non longitudinal electronic product user	1.00 (0.71–1.40)	1.37 (0.99–1.89)	1.25 (0.96–1.62)

^1^ Bone loss around teeth in wave 3 or new cases of gum disease in wave 2 or 3. ^2^ Statistically significant results.

## References

[1] King B.A., Patel R., Nguyen K.H., Dube S.R. (2015). Trends in Awareness and Use of Electronic Cigarettes Among US Adults, 2010–2013. Nicot. Tob. Res..

[2] U.S. Department of Health and Human Services (2016). E-Cigarette Use Among Youth and Young Adults. A Report of the Surgeon General.

[3] Javed F., Kellesarian S.V., Sundar I.K., Romanos G.E., Rahman I. (2017). Recent Updates on Electronic Cigarette Aerosol and Inhaled Nicotine Effects on Periodontal and Pulmonary Tissues. Oral Dis..

[4] Brown L.J., A Brunelle J., Kingman A. (1996). Periodontal status in the United States, 1988–1991: Prevalence, extent, and demographic variation. J. Dent. Res..

[5] Spinell T., DeMayo F., Cato M., Thai A., Lebwohl B., Demmer R.T., Helmerhorst E.J., Green P.H.R. (2018). The association between coeliac disease and periodontitis: Results from NHANES 2009-2012. J. Clin. Periodontol..

[6] Stoltenberg J.L., Osborn J.B., Pihlstrom B.L., Herzberg M.C., Aeppli D.M., Wolff L.F., Fischer G.E. (1993). Association Between Cigarette Smoking, Bacterial Pathogens, and Periodontal Status. J. Periodontol..

[7] Tomar S.L., Asma S. (2000). Smoking-Attributable Periodontitis in the United States: Findings From NHANES III. J. Periodontol..

[8] Rouabhia M., Park H.J., Semlali A., Zakrzewski A., Chmielewski W., Chakir J. (2017). E-Cigarette Vapor Induces an Apoptotic Response in Human Gingival Epithelial Cells Through the Caspase-3 Pathway. J. Cell. Physiol..

[9] Janket S.-J., Baird A.E., Chuang S.-K., Jones J.A. (2003). Meta-analysis of periodontal disease and risk of coronary heart disease and stroke. Oral Surg. Oral Med. Oral Pathol. Oral Radiol. Endodontol..

[10] Gurav A.N. (2012). Periodontitis and Insulin Resistance: Casual or Causal Relationship?. Metab. J..

[11] Lim S.G., Han K., Kim H.A., Pyo S.W., Cho Y.S., Kim K.S., Yim H.W., Lee W.C., Park Y.G., Park Y.M. (2014). Association between insulin resistance and periodontitis in Korean adults. J. Clin. Periodontol..

[12] Saremi A., Nelson R.G., Tulloch-Reid M., Hanson R.L., Sievers M.L., Taylor G.W., Shlossman M., Bennett P.H., Genco R., Knowler W.C. (2005). Periodontal Disease and Mortality in Type 2 Diabetes. Diabetes Care.

[13] Fisher M.A., Taylor G.W. (2009). A Prediction Model for Chronic Kidney Disease Includes Periodontal Disease. J. Periodontol..

[14] Ricardo A.C., Athavale A., Chen J., Hampole H., Garside D., Marucha P., Lash J.P. (2015). Periodontal disease, chronic kidney disease and mortality: Results from the third national health and nutrition examination survey. BMC Nephrol..

[15] Nazir M.A. (2017). Prevalence of periodontal disease, its association with systemic diseases and prevention. Int. J. Health Sci..

[16] Fitzpatrick S.G., Katz J. (2010). The association between periodontal disease and cancer: A review of the literature. J. Dent..

[17] Kamer A.R., Morse D.E., Holm-Pedersen P., Mortensen E.L., Avlund K. (2012). Periodontal inflammation in relation to cognitive function in an older adult Danish population. J. Alzheimers Dis..

[18] Kaye E.K., Valencia A., Baba N., Spiro A., Dietrich T., Garcia R.I. (2010). Tooth loss and periodontal disease predict poor cognitive function in older men. J. Am. Geriatr. Soc..

[19] Kamer A.R., Pirraglia E., Tsui W., Rusinek H., Vallabhajosula S., Mosconi L., Yi L., McHugh P., Craig R.G., Svetcov S. (2015). Periodontal disease associates with higher brain amyloid load in normal elderly. Neurobiol. Aging.

[20] Javed F., Abduljabbar T., Vohra F., Malmstrom H., Rahman I., Romanos G.E. (2017). Comparison of Periodontal Parameters and Self-Perceived Oral Symptoms Among Cigarette Smokers, Individuals Vaping Electronic Cigarettes, and Never-Smokers. J. Periodontol..

[21] Tatullo M., Gentile S., Paduano F., Santacroce L., Marrelli M. (2016). Crosstalk between oral and general health status in e-smokers. Medicine.

[22] Wadia R., Booth V., Yap H.F., Moyes D.L. (2016). A pilot study of the gingival response when smokers switch from smoking to vaping. BDJ.

[23] Huilgol P., Bhatt S.P., Biligowda N., Wright N.C., Wells J.M. (2018). Association of e-cigarette use with oral health: A population-based cross-sectional questionnaire study. J. Public Health.

[24] United States Department of Health and Human Services, National Institutes of Health, National Institute on Drug Abuse, and United States Department of Health and Human Services, Food and Drug Administration, Center for Tobacco Products (2018). Population Assessment of Tobacco and Health (PATH) Study [United States] Public-Use Files.

[25] Hyland A., Ambrose B.K., Conway K.P., Borek N., Lambert E., Carusi C., Taylor K., Crosse S., Fong G.T., Cummings K.M. (2017). Design and methods of the Population Assessment of Tobacco and Health (PATH) Study. Tob. Control.

[26] Stratton K., Kwan L.Y., Eaton D.L., National Academies of Sciences, E. and Medicine (2018). Public Health Consequences of E-Cigarettes.

[27] Shariff J.A., Ahluwalia K.P., Papapanou P.N. (2016). Relationship Between Frequent Recreational Cannabis (Marijuana and Hashish) Use and Periodontitis in Adults in the United States: National Health and Nutrition Examination Survey 2011 to 2012. J. Periodontol..

[28] Saini G.K., Gupta N.D., Prabhat K.C. (2013). Drug addiction and periodontal diseases. J. Indian Soc. Periodontol..

[29] Sundar I.K., Javed F., Romanos G.E., Rahman I. (2016). E-cigarettes and flavorings induce inflammatory and pro-senescence responses in oral epithelial cells and periodontal fibroblasts. Oncotarget.

[30] Willershausen I., Wolf T., Weyer V., Sader R., Ghanaati S., Willershausen B. (2014). Influence of E-smoking liquids on human periodontal ligament fibroblasts. Head Face Med..

[31] Schroeder M.J., Hoffman A.C. (2014). Electronic cigarettes and nicotine clinical pharmacology. Tob. Control.

[32] Tipton D., Dabbous M.K. (1996). Effects of Nicotine on Proliferation and Extracellular Matrix Production of Human Gingival Fibroblasts In Vitro. J. Periodontol..

[33] Lee S.I., Kang K.L., Shin S.I., Herr Y., Lee Y.M., Kim E.C. (2012). Endoplasmic reticulum stress modulates nicotine-induced extracellular matrix degradation in human periodontal ligament cells. J. Periodontal Res..

[34] Malhotra R., Kapoor A., Grover V., Kaushal S. (2010). Nicotine and periodontal tissues. J. Indian Soc. Periodontol..

[35] Pearson J.L., Richardson A., Niaura R.S., Vallone D.M., Abrams D.B. (2012). e-Cigarette Awareness, Use, and Harm Perceptions in US Adults. Am. J. Public Health.

[36] Polosa R., Cibella F., Caponnetto P., Maglia M., Prosperini U., Russo C., Tashkin D. (2017). Health impact of E-cigarettes: A prospective 3.5-year study of regular daily users who have never smoked. Sci. Rep..

[37] Goniewicz M.L., Smith D.M., Edwards K.C., Blount B.C., Caldwell K.L., Feng J., Wang L., Christensen C., Ambrose B., Borek N. (2018). Comparison of Nicotine and Toxicant Exposure in Users of Electronic Cigarettes and Combustible Cigarettes. JAMA Netw. Open.

